# A view of the sustainable computing landscape

**DOI:** 10.1016/j.patter.2025.101296

**Published:** 2025-06-25

**Authors:** Benjamin C. Lee, David Brooks, Arthur van Benthem, Mariam Elgamal, Udit Gupta, Gage Hills, Vincent Liu, Linh Thi Xuan Phan, Benjamin Pierce, Christopher Stewart, Emma Strubell, Gu-Yeon Wei, Adam Wierman, Yuan Yao, Minlan Yu

**Affiliations:** 1 University of Pennsylvania, Philadelphia, PA, USA; 2 Harvard University, Cambridge, MA, USA; 3 Cornell University, Ithaca, NY, USA; 4 Ohio State University, Columbus, OH, USA; 5 Carnegie Mellon University, Pittsburgh, PA, USA; 6 California Institute of Technology, Pasadena, CA, USA; 7 Yale University, New Haven, CT, USA

**Keywords:** computer systems, computer engineering, data centers, artificial intelligence, energy efficiency, environmental sustainability

## Abstract

This article presents a holistic research agenda to address the significant environmental impact of information and communication technology (ICT), which accounts for 2.1%–3.9% of global greenhouse gas emissions. It proposes several research thrusts to achieve sustainable computing: accurate carbon accounting models, life cycle design strategies for hardware, efficient use of renewable energy, and integrated design and management strategies for next-generation hardware and software systems. If successful, the research would flatten and reverse growth trajectories for computing power and carbon, especially for rapidly growing applications like artificial intelligence. The research takes a holistic approach because strategies that reduce operational carbon may increase embodied carbon, and vice versa. Achieving these goals will require interdisciplinary collaboration between computer scientists, electrical engineers, environmental scientists, and economists.

## The bigger picture

The digital world is expanding at an unprecedented pace, driven by the explosive growth of artificial intelligence, data center computing, and networked devices. However, these computing technologies come with an environmental cost that is also growing rapidly but is still poorly understood. These costs arise from the energy-intensive data centers that power artificial intelligence as well as from the manufacture of semiconductors that store and compute on massive datasets. Current trends are unsustainable as artificial intelligence transforms the way we live and work and, consequently, as the demand for computing accelerates.

This article presents an agenda for making computation more sustainable by rethinking how we design, build, and operate digital systems. The agenda is interdisciplinary and spans hardware design, software optimization, energy systems, and economic policy. It seeks to mitigate both embodied carbon, the emissions associated with manufacturing hardware like chips and servers, and operational carbon, the emissions associated with the electricity used to power this hardware. Reducing both types of emissions will require modular hardware organizations that allow greater reuse, energy-efficient data center design and management, and intelligent use of renewable or carbon-free energy. The authors encourage collaboration across disciplines—from computer science and engineering to economics and environmental science—to ensure that technical solutions align with societal goals.

## Teaser

The authors present a holistic research agenda for sustainable computing, addressing the growing carbon footprint of information and communications technology. The agenda is an interdisciplinary perspective that integrates ideas in software design, hardware architecture, renewable energy infrastructure, power and carbon accounting, and economic policy. Collectively, these design and management strategies will reduce the carbon emissions associated with the manufacture and operation of future computer systems, a pressing objective given computing’s rapid growth in emerging applications such as artificial intelligence.

## Introduction

Information and communication technology (ICT) accounts for a surprisingly large share of global greenhouse gas (GHG) emissions—estimates range from 2.1% to 3.9%. To tackle this challenge, the International Telecommunication Union aims for a 45% reduction in ICT emissions by 2030,^1^ aligning with the Paris Agreement’s goal to limit warming to 1.5°C above pre-industrial levels. Meeting the growing demands for computing while achieving these goals will be difficult and costly, requiring rigorous methods that balance sustainability benefits against implementation costs. To succeed, computer scientists, electrical engineers, environmental scientists, and economists must develop an ecosystem for sustainable computing with transformative solutions to computing’s carbon problem. This responds to the call for action from Knowles et al.^2^: computing must end the “digital exceptionalism” that overlooks its carbon footprint due to its contributions to societal productivity and efficiency.

We envision several interlocking research thrusts to address these sustainability challenges for next-generation computer systems. These thrusts take a coordinated approach to hardware and software, designing new processors, servers, and data centers as well as optimizing their deployment for emerging artificial intelligence (AI) applications. These thrusts also take a holistic view of computing’s carbon footprint, reducing embodied carbon from hardware manufacturing via life cycle design strategies and reducing operational carbon from the judicious, timely use of carbon-efficient electricity. We anticipate interesting trade-offs between embodied and operational carbon, as a solution might reduce one type of carbon at the expense of the other. Finally, these solutions must account for the broader economic and policy context to align private initiatives with societal goals.

This article briefly surveys the challenges and opportunities in sustainable computing. It reflects the research priorities of the authors, but the holistic perspective may inspire researchers from diverse intellectual communities—computer science, electrical engineering, industrial ecology, economics, and law—to engage with these questions. We recognize that some of these research questions are becoming qualitatively more challenging due to interest in AI and investment in hyperscale data centers. We also recognize that some of these questions, such as life cycle analysis for hardware, are benefiting from industry attention. This article seeks to place these recent developments in context and encourage greater coordination between these individual research contributions.

## Embodied carbon

Embodied carbon describes emissions associated with computing’s demands on hardware manufacturing and supply chains; the GHG Protocol designates these as scope 3 emissions.^3^^,^^4^^,^^5^ These costs are significant for high-performance computing due to unprecedented data center construction and massive capital investments in graphics processing units and other hardware components for AI. They are also significant for embedded and mobile devices due to high replacement rates and relatively low utilization. Nearly 75% of Apple’s emissions are due to manufacturing.^4^ Billions of devices are expected to come online by 2027, and their embodied carbon may approach one gigaton of CO_2_ per year, exceeding commercial aviation’s footprint.^5^

**Figure 1:**
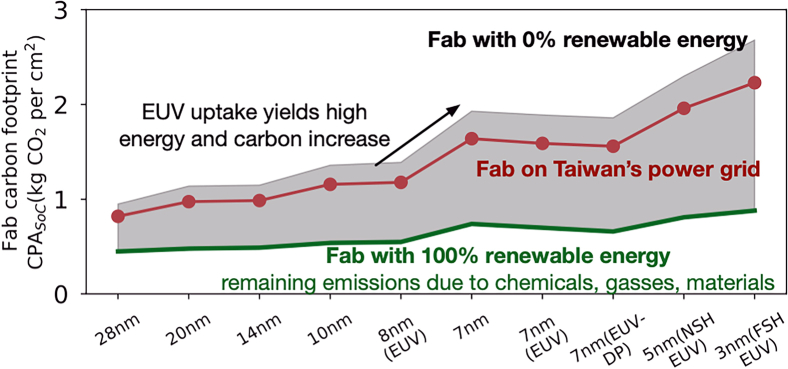
Embodied carbon for semiconductor fabrication Data from industry reports, device characterization.^8^

Semiconductor fabrication’s contributions to global warming are attributed to electricity and gasses used in manufacturing. Electricity use is particularly significant for advanced technology nodes that require extreme ultraviolet lithography (Figure 1). Carbon-free electricity is a meager 6% of the total in Taiwan and South Korea, where most chips are produced, but the Taiwan Semiconductor Manufacturing Company (TSMC) and Korea may increase their use of carbon-free energy to 40% and 20% of their respective totals by 2030.^6^^,^^7^

**Figure 2:**
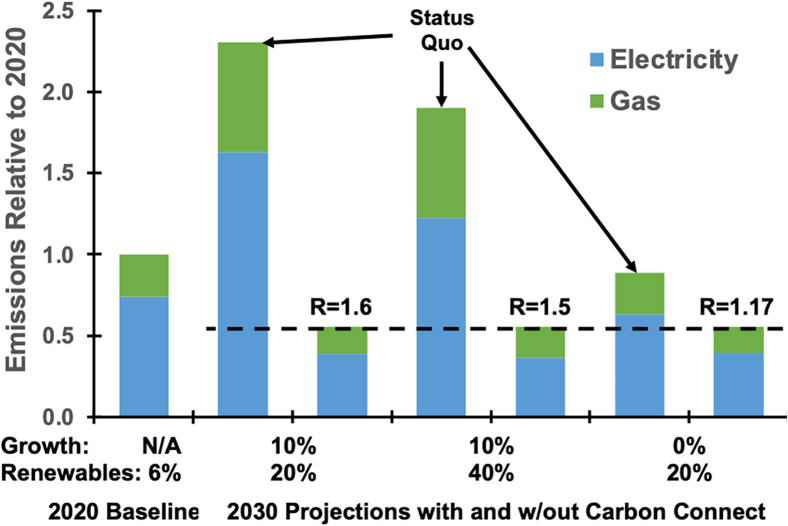
Embodied carbon scenarios that vary fab electricity growth, renewable energy use, characterization, and 3Rs of circular economy

Figure 2 presents several scenarios for embodied carbon. Even under optimistic assumptions where fab demand is unchanged (0%) and the renewable energy supply increases by 20%, the industry will miss its goal of reducing emissions by 45%, as indicated by the dashed line in the figure. This outcome is partially explained by gases, which account for 25% of total emissions and are unaffected by the use of renewable energy. Thus, reducing embodied carbon by 45% requires more aggressive, innovative measures.

Researchers will need to explore several mitigation strategies that arise from the Rs of the circular economy—reduce, reuse, and recycle. Our analysis specifies an “R factor” that estimates the extent to which these Rs are needed to reduce embodied carbon by 45%. For example, R = 1.5 estimates the combined effect of reducing hardware procurement by 33%, reusing hardware 1.5× longer, and recycling 1.5× more hardware relative to 2020 levels. While different combinations are possible, increasing each of the three Rs is essential for the 45% reduction target.

### Reduce

Computer architects should precisely manufacture, provision, and allocate the hardware required for software needs. We need hardware functions that can be designed and implemented separately as small chiplets and then connected with fast networks.^9^ Chiplets are more carbon efficient, as fabs precisely manufacture the required circuits and no more, reducing the silicon area and improving manufacturing yields, which in turn reduces waste and carbon. Moreover, fabs could separate the manufacture of disparate capabilities—compute, memory, and sensors—and use dedicated process flows for each, reducing the number of process steps and associated carbon.

We also need data-center-scale disaggregation, which organizes hardware into collections of network-attached components. Compute nodes would offer many central processing units (CPUs) but little dynamic random access memory (DRAM), whereas memory nodes would offer the reverse. Disaggregation allows servers to independently scale a specific hardware type. “Lego-block” systems with custom core and memory configurations would better balance the system and improve carbon efficiency. Today’s servers provision many DRAMs for capacity but must also inefficiently provision a corresponding number of memory channels and processor sockets even when workloads under-utilize these channels and processors.^10^

### Reuse

Data center operators might replace hardware components based on individual technology advances or failure rates rather than on the fastest evolving or least reliable component, thereby extending the hardware’s average tenure. For example, graphics processing units (GPUs) might refresh at a rate dictated by growing demands for AI workloads, whereas CPUs might refresh at a different rate, tracking demand for general computation. Today, the typical server lifetime is 3–6 years, after which the entire rack is replaced with new hardware. Networking equipment lifetimes are longer, 5 years for switches/routers and 10 years for the fiber cable plant, but periodic and wholesale replacement is still common.

### Recycle

Hardware will require better instrumentation and health models to facilitate an efficient secondary market that disassembles systems into components and sells them for a second life. For instance, heavily used processors from data centers will have very different resale values than lightly used ones from enterprises. Hardware “odometers” could be implemented with immutable, tamper-resistant registers that count operations. For memories, registers might count errors and faults as well as reads and writes. Measures of physical conditions such as power variations, thermal stresses, and humidity will be helpful. These data must be curated by manufacturers, sellers, or third parties so that consumers can intelligently assign value to pre-owned hardware. We draw inspiration from the role that odometers, vehicle history reports, and certified pre-owned designations play in the secondary vehicle market.

## Operational carbon

**Figure 3:**
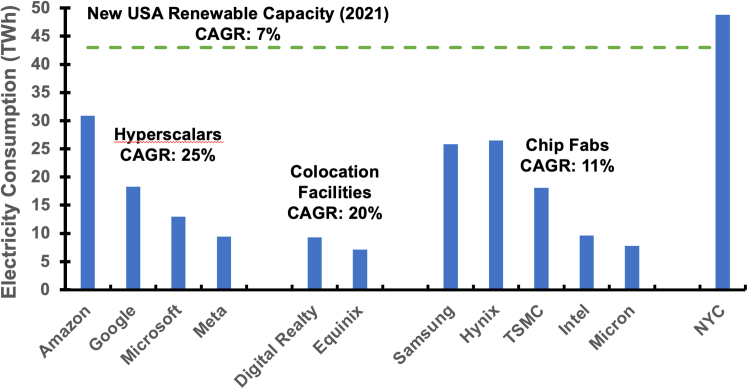
Electricity usage (2021) for data center and fabrication facilities Compound annual growth rate from 2015 to 2021. Corporate sustainability reports and EIA.^4^

Operational carbon describes emissions associated with computing’s electricity use; the GHG protocol designates these as scope 2 emissions. These costs exhibit explosive growth, driven by AI and its myriad applications. Annual ICT energy demand is projected to exceed 100 exajoules, nearly 15% of the world’s energy production.^11^ Electricity use at Google, Meta, and Microsoft grew at a compound annual growth rate (CAGR) of 25% per year from 2015 to 2021, nearly quadrupling. In contrast, US renewable energy investments grew by only 7% per year (Figure 3). In 2021, hyperscale data centers consumed 19 TWh more than in 2020, nearly half of the 44 TWh of new renewable capacity.

**Figure 4:**
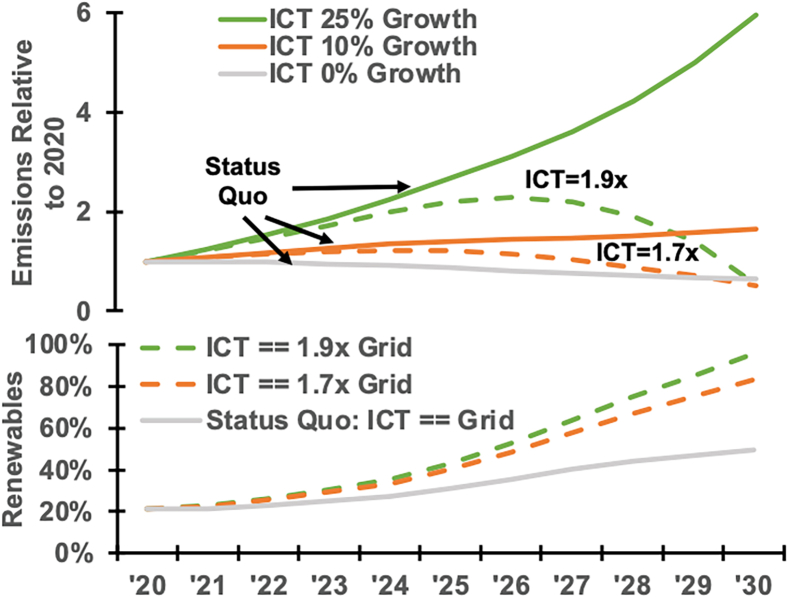
Operational carbon reduction (45% by 2030) achieved via 1.7× higher uptake in ICT renewable electricity compared to the grid

**Figure 5:**
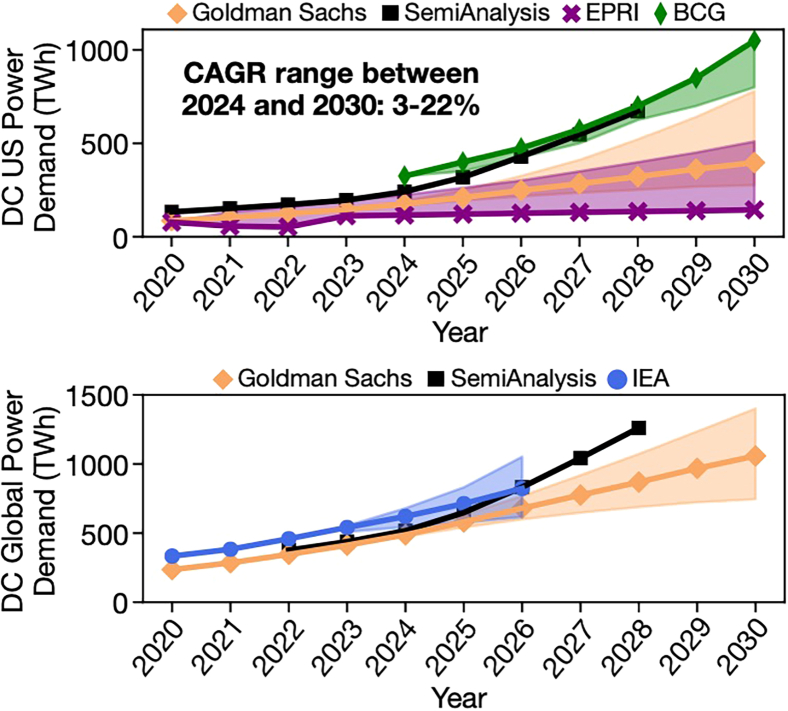
Electricity usage forecasts for data center power in the US and globally Variance in CAGR estimates is significant.

Our analysis highlights the essential role of renewable energy in computing (Figure 4). If renewable energy capacity grows at 10% per year, as forecasted by the US Energy Information Administration (EIA), and computing’s energy demand remains at 2020 levels, carbon emissions would fall by 36%. However, Figure 5 indicates computing’s energy demand may increase by 10%–25% per year based on forecasts by industry groups^11^ and various consultancies.^12^^,^^13^^,^^14^^,^^15^^,^^16^

Carbon-free energy growth would struggle to keep pace. Meeting these demands yet reducing carbon by 45% requires computing to adopt renewable energy at 1.7–1.9× faster than the US average. Nuclear, whether refurbishing existing plants or building small modular reactors, could be a carbon-free alternative. But there is great uncertainty in this pathway, as the nuclear industry must show that it can build capacity on schedule and within budget in the US.

### Demand response

As renewable energy proliferates, sustainable data centers must delay or boost computation based on the availability of carbon-free energy.^17^^,^^18^^,^^19^ Such demand response (DR) requires grid and data center coordination. One interface would use real-time prices to incentivize data centers to modulate energy use, but this departs from today’s contracts that charge based on the amount of power provisioned rather than used. An alternative interface would simply communicate carbon intensity, assuming data centers would modulate demand without compensation.

DR will require hardware and software to trade off performance and power. Ideally, DR frameworks will incentivize participation and guarantee service. Game theory could model system dynamics when users selfishly pursue performance goals. Real-time scheduling and robust machine learning could ensure decisions satisfy diverse obligations. Ultimately, DRs require rethinking conventional wisdom in which data centers constantly compute at peak power to amortize facility and power costs.^20^

### Power modulation

Each user must define and implement multiple operating modes that modulate power when required. Hardware mechanisms will rely on energy proportionality, the idea that power should rise and fall with workload. Energy-proportional hardware is difficult to design because most components have a significant fixed power cost dissipated even at near-zero loads. Decades of research have improved CPUs, but today’s data centers deploy large memory systems and graphics processing units that will need to be designed for energy proportionality.

Software mechanisms will rely on approximate, degraded computing. Online applications implement contingency plans for site events, ensuring varying degrees of service that depend on system availability and downtime. We will explore real-time system design and anytime algorithms to provide a smoother spectrum of trade-offs between quality and power than permitted in today’s systems. Strategies for computational sprinting might allow workloads to dynamically consume additional resources as power budgets permit.^21^

### Intelligent decisions

A cognitive stack could organize power management into a low-level reactive layer and a high-level deliberative layer. An agent monitors software performance and hardware utilization, optimizing power use to achieve performance goals while accounting for data center conditions and competition from other agents. The reactive policy would adjust a processor’s power use based on program phases, while the deliberative policy would ensure that adjustments align with other processors’ policies and data center goals in sustainability, safety, and stability.

The cognitive stack could use multi-agent game theory and reinforcement learning for dynamic decision-making.^22^ Dynamism is crucial because computation varies over time, and allocation decisions in the present should account for the past and anticipate the future. For example, in a repeated game, agents spend tokens for power and learn policies for spending, requesting power, and using hardware. When carbon-free energy is scarce, data centers could offer tokens to jobs that defer their computation or require more tokens from those that do not. How should agents spend tokens to maximize long-term performance when allocations in one time period affect those in an uncertain future? How should data centers price power to achieve sustainability goals?

## Driving applications

AI will drive increasingly rapid growth in computing. Training requires hundreds of thousands of processors that collaboratively consume massive datasets and compute for weeks or months to compute parameter values for a model. Inference requires a rapidly growing number of processors that invoke trained models and respond to user or application prompts, often with ambitious goals for accuracy, response time (i.e., latency), and response rate (i.e., throughput). Efficient AI requires software solutions, such as specialized models that compute equally accurate answers with fewer calculations,^23^ and hardware solutions, such as application-specific integrated circuits, that reduce the cost of each calculation.

Advances in AI are enabled by scaling deep models and their training data,^24^ which impacts sustainability.^25^^,^^26^ Benchmarking AI’s carbon footprint would help researchers identify the most pressing challenges.^27^ An integrated hardware-software perspective will be particularly helpful as researchers explore the net impact of custom hardware,^28^ which reduces operational carbon through energy efficiency but increases embodied carbon through semiconductor manufacturing.

Sustainable AI hinges on its responsiveness to the varying availability of data, hardware, and electricity. We will need to design, train, and deploy AI models that offer performance and efficiency on a broad spectrum of hardware platforms. Such models would not only ensure backward compatibility for and equity of access to AI features, but they might also slow the rate of hardware refreshes. How can we develop models and platforms that remain relevant over longer periods and better amortize the carbon costs of model training?

There is a complementary need for programmable, reconfigurable hardware that supports a broad spectrum of AI workloads. Such processors would allocate precisely the hardware required for data processing, training, or inference, consuming energy in proportion to utilization. Instead of designing static AI accelerators, how can we develop flexible, general processors that are relevant for large classes of AI computation and better amortize embodied carbon from semiconductor fabrication?

If successful, this research agenda will reverse current trends and permit advanced AI with lower carbon costs. Google consumes 1.5–2.3 TWh for AI, 10%–15% of its total energy use.^29^ Meta attributes 30% of its AI energy to data processing, 30% to model training, and 40% to inference.^27^ Studies for BLOOM’s 176B-parameter language model, a GPT-3 replica, are also alarming. Training uses 433 MWh and emits 25 T-CO_2_e, whereas inference uses 914 KWh and emits 19 kgs-CO_2_e per day, assuming 558 requests per hour.^30^

## Carbon accounting

Research in reducing the environmental impact of AI will only be effective with the right metrics and accurate datasets. Measuring embodied carbon requires standardized methods across the industry’s many companies and organizations, as well as extensible methods that accommodate new and emerging technologies. Measuring operational carbon requires scalable telemetry from large, distributed systems, such as hyperscale data centers, that track resource and power utilization. Transparency will be key to building trust and confidence.

Modeling embodied carbon from semiconductor manufacturing is difficult because complex fabrication processes are evolving to accommodate emerging technologies such as nanomaterials,^31^ photonic devices,^32^ and heterogeneous integration.^33^ Yet, we are optimistic given recent advances in technology models and life cycle analyses.^34^^,^^35^ Moreover, the manufacture of “new” technologies actually leverages many existing process flows. By mixing and matching steps in mature flows—lithography, metal and oxide deposition, etching, thermal annealing, etc.—we might estimate carbon for flows not yet in production. For example, the first monolithic 3D process flow that integrates next-generation transistors and resistive random access memory (RRAM) re-orders existing steps and adds one new step.^36^

Operational carbon depends on the energy consumed, and we need energy profilers for individual tasks, helping operators track usage and guide management. System telemetry will be combined with grid telemetry, but estimating electricity’s carbon intensity is non-trivial. The marginal emission rate, which depends on recently activated generation sources, may overstate carbon because data centers often receive credits from their renewable energy investments and because grids often transfer energy across regional boundaries.

Telemetry lays the foundations for attribution, which assigns responsibility for carbon to individual pieces of computation.^37^ A task’s operational carbon depends on its share of data center overheads. Estimating a task’s share of embodied carbon requires sophisticated analysis because tasks share servers and each task uses heterogeneous mixes of hardware. Game theory and the Shapley value may provide frameworks for fair attribution.^38^

We require reliable, harmonized, and transparent methods for carbon accounting. Data centers’ energy use and emissions are verifiable by using the EPA’s carbon statistics for power plants and measuring energy for hardware components. Semiconductor fabrication’s energy use is more difficult to verify but could leverage published sustainability reports and datasets. Open-source models for life cycle assessment (LCA) methods would lay the foundations for improving analysis and engaging stakeholders.^39^ Although computing does not yet have such foundations, the EPA and California have set standards to reduce emissions from fuels using open-source tools.^40^

## Energy economics

Research in computing must be cognizant of the broader societal landscape. External factors may make some solutions more practical than others or may provide opportunities to amplify or accelerate anticipated benefits. Economics and policy shape pathways to carbon-efficient computing. Governments might introduce carbon trading or incentives for low-carbon energy, while the private sector could implement offset programs, leading to renewable energy contracts and credits. DR will need sophisticated markets that price electricity at its true marginal cost, encouraging users to schedule computation accordingly. Although there is extensive literature on low-carbon policies for other industries,^41^ economic analysis for computing remains relatively unexplored. Data centers, often the largest grid consumers, must understand how their net-zero operations affect other consumers and society.

Given the unpriced environmental externality of carbon,^42^ one might ask if society is computing too much. What is the optimal amount of computing? Will more efficient algorithms and systems drive demand for new applications, increasing overall carbon emissions? Prior research suggests that as technology becomes more efficient, its use increases, producing rebound effects that range from 10% to 40%, reducing but not eliminating energy savings.^43^ However, these effects have not been studied for computing.

We need to estimate three types of rebound effects. First, direct effects occur when lower costs increase technology use. Data centers likely exhibit strong direct effects, as more efficient processors lead to data centers with more processors. Second, indirect effects arise when lower costs increase the use of other technologies. This requires understanding the interplay between hardware components; more efficient processors may require more memory. Finally, macroeconomic effects arise when lower costs encourage new applications. Efficient processors may scale the use of large AI models for tasks like conversational bots.

## Conclusion

Computing is at a moment of profound opportunity and promise. Emerging applications are driving unprecedented growth for systems that offer scalable performance and environmental sustainability. Despite advances toward net-zero carbon emissions, the industry’s gross energy usage continues to rise, outpacing new energy installations and renewable energy deployments. A shift toward sustainability could transform how systems are manufactured, allocated, and consumed, leading to a more responsible approach to new technologies.

As researchers establish new standards for carbon accounting, they may influence policy and legislation. An interdisciplinary community dedicated to sustainable computing is needed to train the next generation of innovators in technology, economics, and policy. Partnerships between academia and industry would accelerate the adoption of sustainable practices. Only by working together can we create holistic solutions that sustain advances in computation, revolutionizing the way we live and work for decades to come.

## Acknowledgments

This work is supported by the National Science Foundation Expedition in Computing (CCF-2326605, 2326606, 2326607, 2326608, 2326609, 2326610, and 2326611). Any opinions, findings, conclusions, or recommendations expressed in this material are those of the authors and do not necessarily reflect the views of this sponsor.

## Author contributions

Conceptualization overall, B.C.L. and D.B.; conceptualization for embodied carbon, D.B., G.H., V.L., and G.-Y.W.; conceptualization for operational carbon, B.C.L., M.E., L.T.X.P., C.S., and A.W.; conceptualization for driving applications, B.P. and E.S.; conceptualization for carbon accounting, U.G., Y.Y., and M.Y.; conceptualization for energy economics, A.v.B. and B.C.L.; data science for embodied and operational carbon, D.B., G.-Y.W., G.H., and M.E.; writing – original draft, B.C.L., A.v.B., V.L., E.S., G.-Y.W., M.Y., and Y.Y.; writing – review & editing, B.C.L. and B.P.

## Declaration of interests

B.C.L. is a visiting/consulting scientist at Google. A.W. is a member of the advisory boards for Freeflow Ventures, Verrus, and Virtualitics.
